# Sinonasal small-cell carcinoma combined concurrently with small-cell lung carcinoma: case report and literature review

**DOI:** 10.3389/fimmu.2025.1457907

**Published:** 2025-01-29

**Authors:** Rencui Quan, Ling Han, Shihai Wu, Xianming Li

**Affiliations:** ^1^ Jinan University, Guangzhou, China; ^2^ Department of Radiation Oncology, Shenzhen People’s Hospital (The Second Clinical Medical College, Jinan University, The First Affiliated Hospital, Southern University of Science and Technology), Shenzhen, Guangdong, China; ^3^ Department of ENT, Shenzhen People’s Hospital (The Second Clinical Medical College, Jinan University, The First Affiliated Hospital, Southern University of Science and Technology), Shenzhen, Guangdong, China

**Keywords:** sinonasal small cell neuroendocrine carcinoma, small cell lung carcinoma (SCLC), immune checkpoint inhibitors, PD-L1, maintenance treatment

## Abstract

Sinonasal small-cell neuroendocrine carcinoma (SCNEC) is an uncommon malignant epithelial neuroendocrine tumor in the sinonasal cavity that often presents in isolation and rarely occurs in synchronous fashion with small-cell lung carcinoma (SCLC). Here, we present a case of a 65-year-old man diagnosed with SCNEC concurrently combined with SCLC. He received first-line platinum-doublet chemotherapy combined with durvalumab, followed by radiotherapy to thoracic as well as head and neck regions. The follow-up computed tomography and magnetic resonance imaging showed a complete response according to Response Evaluation Criteria in Solid Tumors criteria until 9 June 2024. This case highlights the need for accurate diagnostic characterization of primary lesions and the need to formalize treatment paradigms using chemotherapy, radiation therapy, immunotherapy with immune checkpoint inhibitors, and targeted prophylactic cranial irradiation.

## Introduction

Sinonasal small-cell neuroendocrine carcinoma (SCNEC) is a poorly differentiated malignant epithelial neuroendocrine tumor in the sinonasal cavity (1). The nasal cavity and septum (32.5%) was the most common site of involvement, followed closely by the ethmoid sinus (31.3%) (1). The fifth edition of the classification of Head and neck Tumors published by the World Health Organization defined well-differentiated epithelial neuroendocrine neoplasms as neuroendocrine tumors (NET) and poorly differentiated neuroendocrine neoplasms (neuroendocrine carcinomas, NECs). The latter are further subtyped based on cytomorphological characteristics into small-cell neuroendocrine carcinomas and large cell neuroendocrine carcinomas (2). Immunohistochemical biomarkers, including (but not limited to) markers of neuroendocrine differentiation (insulinoma-associated protein 1, chromogranin-A, and synaptophysin), cytokeratin, and Ki-67, help to define the neuroendocrine nature accurately. Some scholars have explored the difference in SCNEC from other types of undifferentiated carcinoma in the head and neck region by neuroendocrine immunohistochemical indicators combining the genetic profile. They have confirmed the pattern of expression of cytokeratin and neuroendocrine components, and genome-wide copy number profiling helps to distinguish the diagnosis. The cytokeratin and neuroendocrine marker expression pattern is positive, and whole chromosome segments carry high-level gains and losses in sinonasal SCNEC (3). Sinonasal SCNEC is a rare and aggressive carcinoma that accounts for only 5% of all sinonasal malignancies, and standard treatment is lacking (4). Kao and colleagues reported that the 5-year overall survival (OS) was 21.1% in patients with sinonasal SCNEC (5). Sinonasal SCNEC makes up a very small percentage of extrapulmonary small-cell carcinoma (ESCC). According to the research encompassing 5,747 patients diagnosed with ESCC from 2006 to 2014, the result revealed that the most common site of diagnosis was the genitourinary subsites (43%), followed by gastrointestinal (27%), gynecologic (17%), head and neck (11%), and breast (2%) (6).

Given the scarcity of sinonasal SCNEC cases, the management paradigm is derived primarily from the treatment strategies for SCLC or the standard treatment tailored to the site of primary disease. For limited-stage SCLC, concurrent chemotherapy along with thoracic radiotherapy is first-line treatment because existing randomized controlled trials (RCTs) do not endorse surgical resection as a treatment option except for patients with very early-stage disease (7–11). For extensive-stage SCLC, four to six cycles of platinum plus etoposide have been the preferred first-line treatment (12–14). Recently, the addition of immunotherapy has established new standards for first-line therapy in extensive-stage SCLC, which improved the overall survival dramatically (15–17). Two phase III RCTs that investigated the role of thoracic radiotherapy in extensive-stage SCLC demonstrated that patients who received thoracic radiotherapy achieved significantly longer survival than those who received chemotherapy alone (median OS 17 vs. 11 months; 5-year survival 9.1% vs. 3.7%, respectively; *P* = 0.041) (18, 19). Prophylactic cranial irradiation (PCI) reduces the risk of symptomatic brain metastases significantly compared with observation and leads to a longer median survival in SCLC of any stage (20, 21). PCI has been shown to lengthen survival in SCLC. However, its role in sinonasal SCNEC remains unclear because studies have indicated that brain metastasis occurs less frequently in patients with sinonasal SCNEC compared with those with SCLC (6, 22). However, a notable exception indicates that ESCC originating from the head and neck region is associated with a higher incidence of brain metastasis, thereby justifying the addition of PCI in such cases (23).

## Case presentation

**Figure 1:**
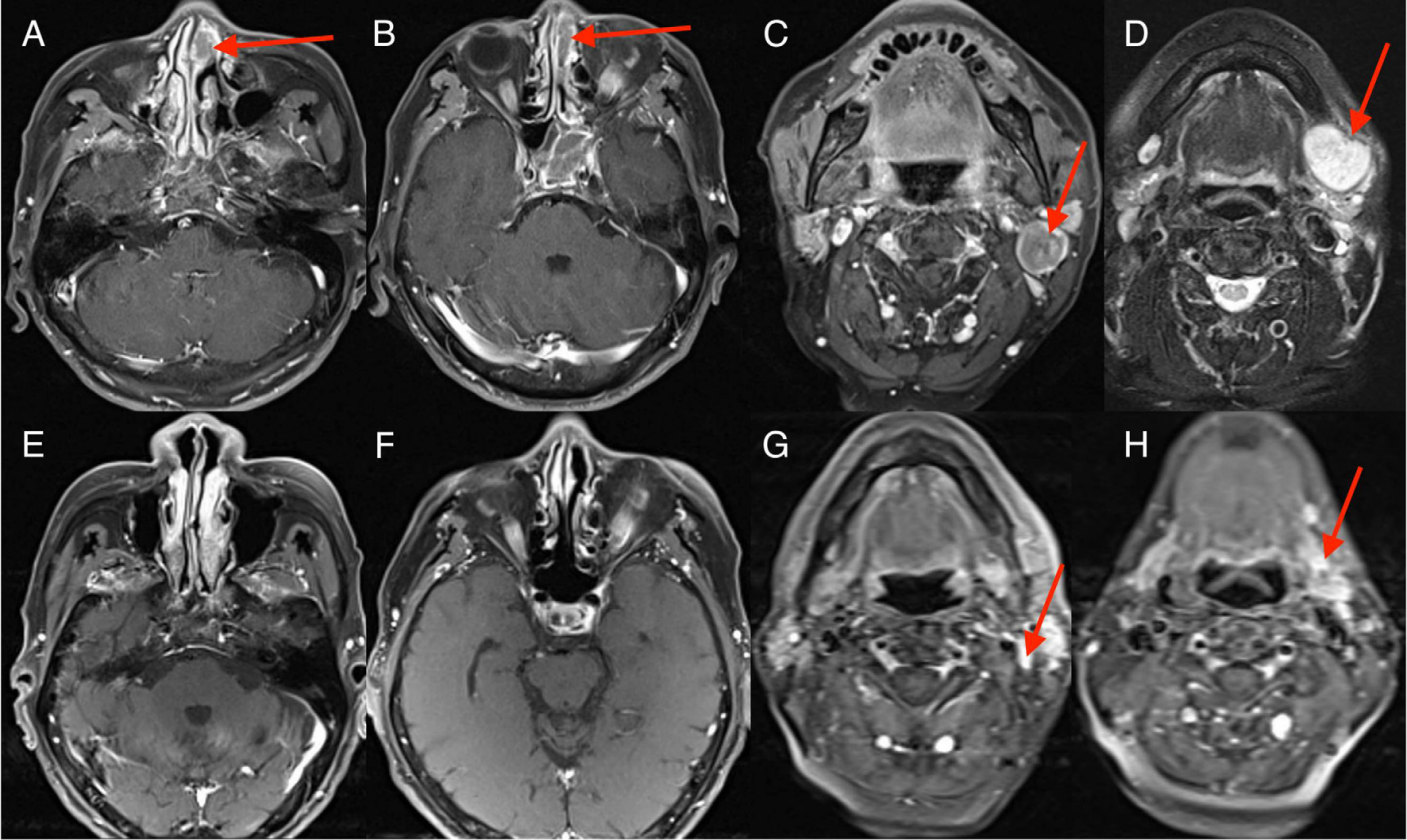
Imaging changes during treatment. Magnetic resonance imaging (MRI) images demonstrating diagnosis, treatment responses. [**(A–D)** Sinonasal squamous cell carcinoma (SCC) was first diagnosed (15 July 2021); MRI scan showed a 3.7 cm × 2.0 cm soft tissue lesion in sinonasal and enlarged lymph node in the left upper neck region. **(E–H)** reduced tumor during four cycles of EP and durvalumab (October 15, 2021); MRI scan showed sinonasal SCC disappeared and neck lymph nodes reduced.].

**Figure 2:**
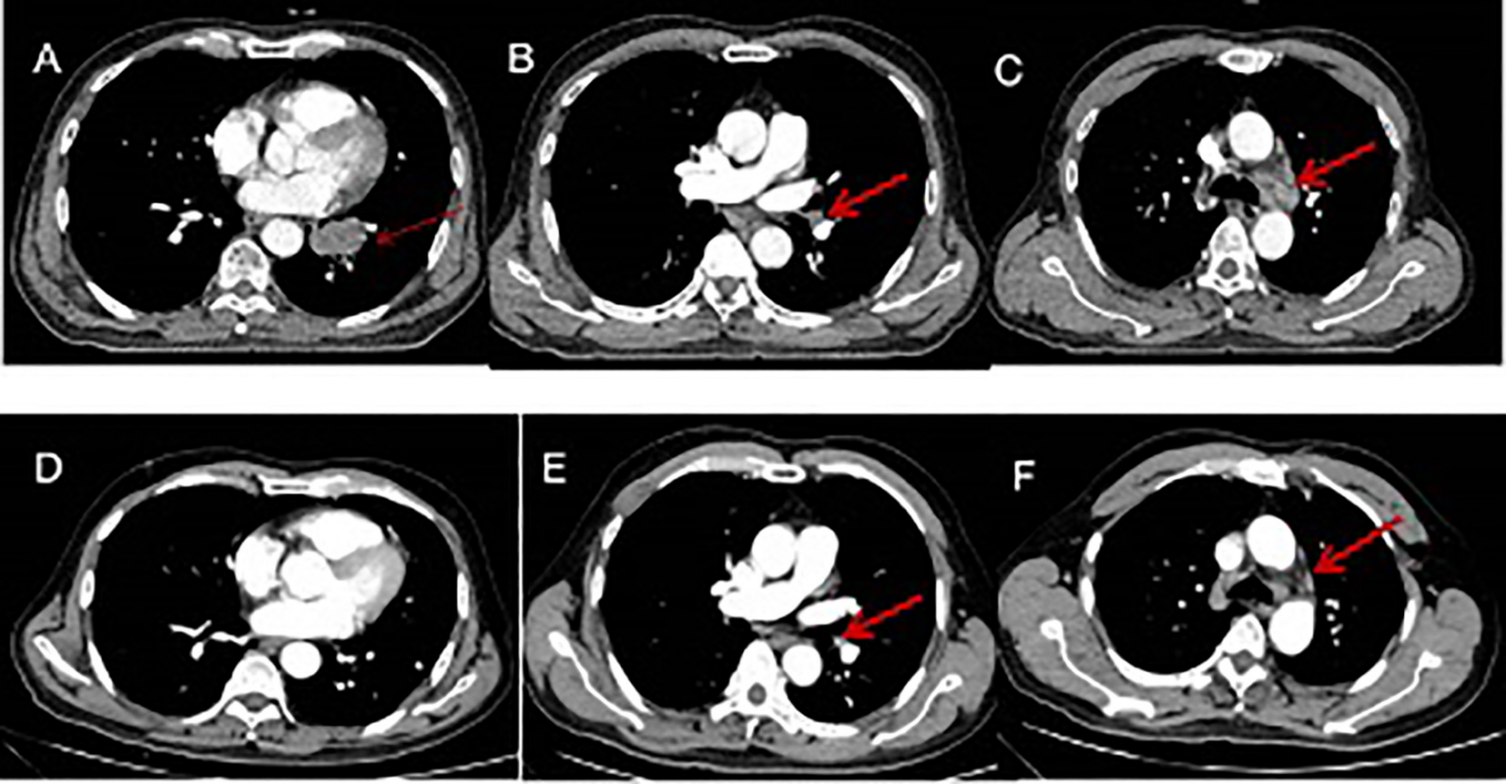
Imaging changes during treatment. CT images demonstrating diagnosis, treatment responses. [**(A–C)** Small-cell lung cancer was first diagnosed (15 July 2021), CT scan showed a 3.7 cm × 2.1 cm pulmonary nodule metastases and enlarged mediastinal lymph nodes. **(D–F)** reduced tumor during four cycles of EP and durvalumab (15 October 2021), CT scan showed left lower lung tumor disappeared and mediastinal lymph nodes reduced.

**Figure 3:**
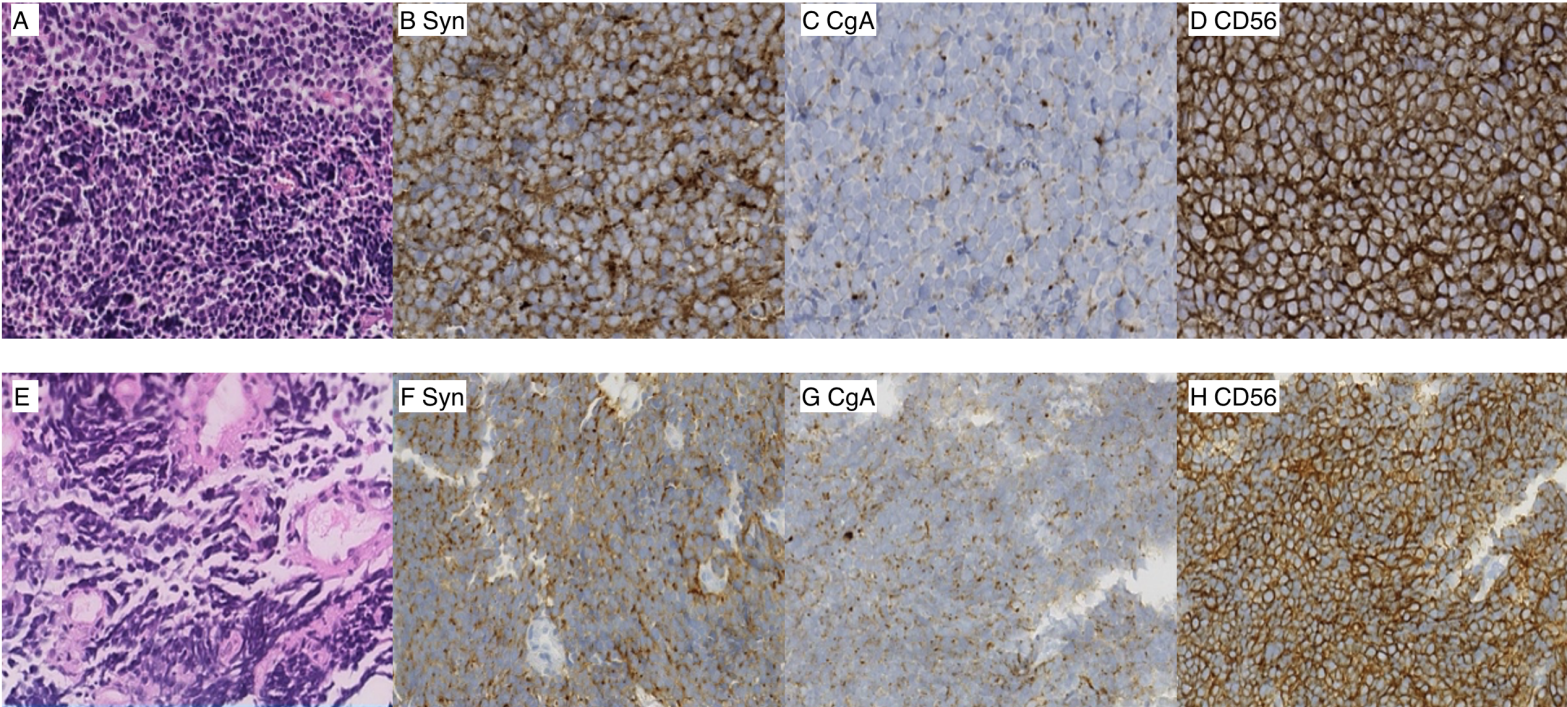
Photomicrographs of the sinonasal lesion **(A–D)** (magnification ×40). **(A)** Hematoxylin and eosin (H&E) staining, **(B)** immunohistochemistry of synaptophysin, **(C)** chromogranin A, **(D)** neural cell adhesion molecule, CD56. Photomicrographs of the lung lesion **(E–H)** (magnification ×40). **(E)** Hematoxylin and eosin (H&E) staining, **(F)** synaptophysin, **(G)** chromogranin A, **(H)** neural cell adhesion molecule, CD56.

On 14 July 2021, a 65-year-old man presented with a 1-month history of nosebleed and a progressively growing painless left-neck mass. The patient had not received any treatment prior to this visit to our clinic. He denied associated hoarseness, otalgia, dysphagia, blurred vision, cough, dyspnea, or hemoptysis. Physical examination revealed the presence of enlarged lymph nodes, one on the left submandibular and another on the upper neck, which had a diameter of 3 cm × 3 cm. Pain was not felt upon palpation. The patient had no significant past medical history. He lacked a history of tobacco smoking and had no familial history of cancer. Laboratory tests revealed an increased level of neuron-specific enolase (NSE: 20 ng/ml), which exceeded the normal reference range of 16 ng/ml. Routine analyses of blood and urine, liver enzymes, renal function, and serologic tests (including human immunodeficiency virus, hepatitis B virus, and syphilis) were normal. Contrast-enhanced magnetic resonance imaging (MRI) of the nasal cavity and sinuses and neck indicated an enhanced soft-tissue lesion in the left nasal and ethmoidal cellules measuring 3.7 cm × 2.0 cm at maximum cross-section and neck enlarged lymph nodes in the neck (**Figures 1A–D**). Contrast-enhanced computed tomography (CT) of the chest revealed a solid pulmonary nodule in the left lower lobe measuring 3.7 cm × 2.1 cm at maximum cross-section, with multiple enlarged mediastinal lymph nodes (**Figures 2A–C**). Fine-needle aspiration of the mass in the left nasal region and left lower lung showed high-grade NEC. The tumor cells exhibited a uniform morphology, closely resembling that of lymphocytes. These cells were approximately 2–3 times larger than mature lymphocytes. Morphologically, the tumor cells demonstrated scant cytoplasm and granular chromatin. The cell shape ranged from oval to spindle-shaped, with a notable absence of nucleoli. In certain areas, prominent crush artifacts were observed (which is an indicative feature for small-cell carcinoma). The immunohistochemistry section exhibited neuroendocrine differentiation, as indicated by strong positivity for synaptophysin and cluster of differentiation-56 and patchy positivity for chromogranin A. Meanwhile, the tumor cells had a Ki-67 proliferation index of 95% and were negative for the squamous-cell marker p63 and adenocarcinoma marker cytokeratin-7 (**Figures 3A–H**). Examination was positive for programmed death-ligand 1 [PD-L1; combined positive score (CPS) ≥20]. Enhanced MRI of the brain revealed no indications of metastatic lesions. Contrast-enhanced CT of the abdomen and bone scintigraphy demonstrated an absence of metastasis. Thus, the preoperative clinical stage was defined as stage IVa in sinonasal small-cell carcinoma (cT4N3bM0) and stage IIIa in SCLC (cT2N2M0) according to the eight edition of the American Joint Committee on Cancer staging system.

## Treatment and outcome

The patient received chemotherapy and immunotherapy with six cycles of cisplatin, etoposide, and durvalumab from 29 July to 30 November 2021. The standard dose was used: cisplatin (60–80 mg/m^2^) on day 1; etoposide (100–120 mg/m^2^) on days 1, 2, and 3; and durvalumab (1500 mg) on day 1 of every three-week cycle. Contrast-enhanced MRI showed the soft-tissue lesion in the left nasal region and ethmoidal cellules had disappeared, and enlarged lymph nodes in the neck region reduced after four cycles of treatment with etoposide, cisplatin, and durvalumab (**Figures 1E–H**). CT of the chest showed the tumor in the left lower lung to have disappeared, and enlarged mediastinal lymph nodes reduced after four cycles of treatment with etoposide, cisplatin, and durvalumab (**Figures 2D–F**). The patient received an additional two cycles of treatment with etoposide, cisplatin, and durvalumab due to his significant response and tolerance. Then, the patient received the maintenance treatment with durvalumab until April 2022. MRI revealed residual enlarged lymph nodes in the neck and mediastina. Subsequently, the patient received thoracic radiotherapy from 13 April to 4 May 2022. The standard radiation dose was 45 Gy b.i.d. in 30 fractions over 3 weeks to the primary tumor region and involved mediastinal lymph nodes. Shortly afterwards, he received radiotherapy to the head and neck between 9 May and 20 June 2022. The standard radiation doses were 63 Gy in 30 fractions to the primary tumor region, 66 Gy in 30 fractions to the residual enlarged lymph nodes in the neck, and 50.1 Gy in 30 fractions to the preventive neck lymph node area (levels Ib, II_IV) over 7 weeks. The patient tolerated this treatment well. Follow-up CT of the lung and MRI of the head and neck showed a complete response at the end of radiotherapy until now. Considering the high aggressiveness of small-cell carcinoma and the medication donation policy, the patient received the maintenance treatment of durvalumab for 2 years in total until 1 August 2023. He refused to receive PCI due to his satisfaction with the complete remission of the primary tumor.

**Figure 4:**
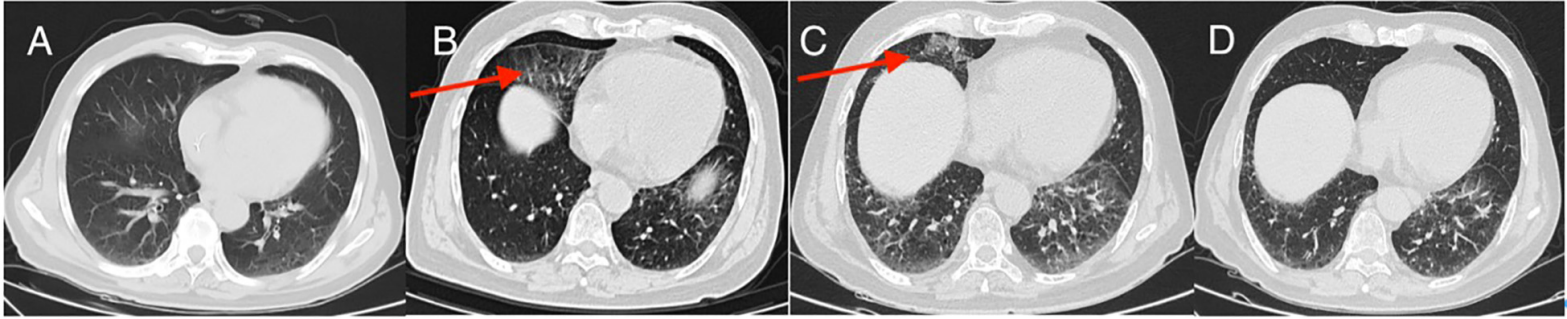
CT manifestations of pneumonia **(A–D)**; **(A)** no signs of pneumonia after three cycles of immunotherapy; **(B)** immune pneumonia after 10 cycles of immunotherapy (red arrow); **(C)** immune pneumonia after 16 cycles of immunotherapy (red arrow); **(D)** immune pneumonia after 10 cycles of immunotherapy disappeared after 20 cycles of immunotherapy.

From the initial hospital admission until 9 June 2024, he had regular monitoring encompassing routine analyses of blood, liver and kidney functions, myocardial enzymes, and thyroid function. During this monitoring period, these tests consistently indicated no significant abnormalities, and the patient suffered only mild immune pneumonia (**Figures 4A–D**) and did not develop any other immune system-related symptoms.

## Discussion

This case represents an exceptionally rare instance of SCNEC affecting the nasal and ethmoidal cells. In this case, the classic and distinctive histologic findings observed under staining (hematoxylin and eosin) were sufficient to make the diagnosis. Furthermore, the positive results for synaptophysin and chromogranin provided additional support for this diagnosis. In terms of pathological morphology, the microscopic features of a sinonasal lesion and lung neoplasm were identical. With respect to imaging feature, the nasal cavity and sinuses are not typical metastatic sites for SCLC. Conversely, the sinonasal small-cell carcinomas are predominantly locally advanced tumors with a relatively low risk of distant metastasis. Van der and colleagues showed that, among 115 cases of small-cell carcinomas of the nasal cavity and sinuses, only 3.1% exhibited distant metastasis (24). Furthermore, metastatic lung cancer typically manifests in the peripheral zones of the lungs, with multiple nodules. Consequently, distinguishing whether a patient’s condition is a result of tumor metastasis or two primary lesions is difficult. After comprehensive discussion, we have decided to treat the patient based on the approach for extensive-stage SCLC: immunotherapy combined with cisplatin plus etoposide (15–17). The largest study of ESCC reported to date demonstrated that chemoradiation yielded the longest survival (22). The study cohort comprised 5,747 patients, who exhibited a median OS after ESCC in the head and neck region of 23.64 months, and chemoradiation improved the OS (6). The OS of patients received radiotherapy for the head and neck (with or without chemotherapy) was twice that of patients receiving chemotherapy alone. Head and neck subsites were associated with improved OS, which hinted that a radical dose of radiotherapy was beneficial to survival. The addition of surgery to radiotherapy and chemotherapy could not improve survival in patients (6). Similarly, another study reported that 10 patients with primary SCNEC had a median OS of 24.7 months (range: 5.7–247.4) months (25). A meta-analysis suggested that the 5-year disease-specific survival rate of SCNEC was 46.1% after comprehensive treatment including surgery, radiotherapy, and chemotherapy (24). Overall, the combination of radiotherapy and chemotherapy is the modality employed most frequently for SCNEC.

PCI has been recommended as the standard treatment for patients with stage IV SCLC aged <75 years old, performance score 0–2 and who have no progression after first-line chemotherapy (26). However, studies have shown that ESCC carries a lower prevalence of early death and brain metastasis compared with that of SCLC (27–29). Daniel et al. (28) reported that only one patient of 18 patients diagnosed with ESCC had an isolated brain metastasis. Similarly, our patient did not receive PCI, and there were no observable signs of brain metastasis even after 3 years. Nevertheless, the efficacy of PCI for therapy of SCNEC has not been shown, and data are insufficient to give PCI routinely (22, 23). However, a retrospective analysis concluded that anti-PD-1/PD-L1 therapy failed to reduce the risk of developing brain metastases in patients with small-cell carcinoma (30). Hence, the absence of PCI in our case is controversial.

Immunotherapy is a promising approach for SCLC. A trend for higher response rates to the standard chemotherapy with/without radiotherapy has been documented (16, 17). Salhab M and colleagues showed that patients with ESCC in the PD-L1 positive group exhibited a superior overall response rate and median OS compared to those in the PD-L1 negative group (31). Authors documented that 12 of 34 cases (35%) with PD-L1 had a CPS score ≥1, and two cases had a CPS score >80. Our patient was positive for PD-L1 (CPS ≥20). During follow-up, our patient exhibited grade-I radiation pneumonitis in the 16th cycle of durvalumab, devoid of clinical symptoms such as cough or sputum. Fortunately, the pneumonia resolved spontaneously in the 26th immunotherapy cycle without any intervening treatment. Wang et al. (32) conducted the most extensive and comprehensive meta-analysis on treatment-related adverse events associated with immune checkpoint inhibitors. They revealed that PD-L1 inhibitors exhibited a higher safety profile compared with PD-1 inhibitors, with a lower prevalence of adverse events of grade ≥3. That observation could be attributed to the presence of an additional PD-1 ligand, PD-L2, which potentially sustains a certain level of checkpoint signaling.

The coexistence of SCNEC and squamous cell carcinoma (SCC) is relatively rare, and the incidence of brain metastasis is not known. This particular case offers a new perspective on potential treatment strategies. The integration of immunotherapy with a platinum-based regimen plus local radiotherapy can lengthen survival rates for patients with SCNEC and SCC, thereby presenting a novel therapeutic avenue.

## Data Availability

The original contributions presented in the study are included in the article/supplementary material. Further inquiries can be directed to the corresponding author.
